# Incorporation of Remote PM_2.5_ Concentrations into the Downscaler Model for Spatially Fused Air Quality Surfaces

**DOI:** 10.3390/atmos11010103

**Published:** 2020-01-15

**Authors:** Brett Gantt, Kelsey McDonald, Barron Henderson, Elizabeth Mannshardt

**Affiliations:** 1 Office of Air Quality Planning and Standards, Environmental Protection Agency, Research Triangle Park, NC 27711, USA; 2 Department of Psychology & Neuroscience, Duke University, Durham, NC 27708, USA

**Keywords:** air quality, PM_2.5_, fused surface, IMPROVE, Downscaler

## Abstract

The United States Environmental Protection Agency (EPA) has implemented a Bayesian spatial data fusion model called the Downscaler (DS) model to generate daily air quality surfaces for PM_2.5_ across the contiguous U.S. Previous implementations of DS relied on monitoring data from EPA’s Air Quality System (AQS) network, which is largely concentrated in urban areas. In this work, we introduce to the DS modeling framework an additional PM_2.5_ input dataset from the Interagency Monitoring of Protected Visual Environments (IMPROVE) network located mainly in remote sites. In the western U.S. where IMPROVE sites are relatively dense (compared to the eastern U.S.), the inclusion of IMPROVE PM_2.5_ data to the DS model runs reduces predicted annual averages and 98th percentile concentrations by as much as 1.0 and 4 μg m^−3^, respectively. Some urban areas in the western U.S., such as Denver, Colorado, had moderate increases in the predicted annual average concentrations, which led to a sharpening of the gradient between urban and remote areas. Comparison of observed and DS-predicted concentrations for the grid cells containing IMPROVE and AQS sites revealed consistent improvement at the IMPROVE sites but some degradation at the AQS sites. Cross-validation results of common site-days withheld in both simulations show a slight reduction in the mean bias but a slight increase in the mean square error when the IMPROVE data is included. These results indicate that the output of the DS model (and presumably other Bayesian data fusion models) is sensitive to the addition of geographically distinct input data, and that the application of such models should consider the prediction domain (national or urban focused) when deciding to include new input data.

## 1. Introduction

Several recent epidemiology and exposure studies linking air pollution concentrations to health effects have transitioned from using composite monitors to continuous air quality surfaces [1-3], necessitating the need for additional refinement and evaluation of the methods used to generate these surfaces. Conceptually, continuous surfaces are an improvement because they capture the gradients of exposure fields that composite monitors neglect. These studies have generated air quality surfaces by fusing monitoring data with chemical transport models, satellite-based estimates, land surface models, and/or population density estimates [4-7]. In addition to the different types of input data, several different statistical models have been used for the fusion calculations. For a recent review of the different input data and statistical models used for data fusion of ambient air pollution, see Shaddick et al. [8]. Few of these past studies, however, describe the sensitivity of these surfaces to the spatial coverage of observed input data used to derive the fields.

In recent years, the United States Environmental Protection Agency (EPA) has used a Bayesian spatial downscaling fusion model called the Downscaler (DS) model [9] to create air quality surfaces of 24-h average PM_2.5_ concentrations of the contiguous U.S. for use in the draft Policy Assessment of the National Ambient Air Quality Standards (NAAQS) for Particulate Matter [10] and the Centers for Disease Control and Prevention’s (CDC) National Environmental Public Health Tracking Network [11]. The DS model also has the potential to be utilized in other air quality applications, including data fusion for EPA’s AirNow or citizen scientists using low-cost sensors to monitor pollution levels in their region.

## 2. Methods

As inputs to the DS model, we used both point-level monitoring data from EPA’s Air Quality System (AQS) network and gridded model output from the Community Multiscale Air Quality (CMAQ) model [12]. The two data sources combine the accuracy of measurement data and the spatial coverage of CMAQ predictions to create an accurate air quality surface with realistic gradients. The DS model uses a spatially varying weighted model to derive spatial covariate terms for the CMAQ grid cells surrounding the monitoring station. A local smoothed surface is generated via a mean-zero Gaussian Markov random field with an exponential covariance structure, which then implicitly relates the monitored values to the CMAQ modeled output within the neighboring grid cells via random weights that are spatially varying according to an exponential kernel with an empirically estimated decay parameter [9]. A spatial regression relationship between monitoring station point-level measurements and gridded model output is then determined by regressing monitoring data onto the derived CMAQ regressors. Applying the model on the spatially complete gridded CMAQ surface allows for point-level predictions at locations where a monitor may not exist, hence downscaling. The regression relationship is applied separately for each time step of data, and can be expressed in a generalized equation as follows:

(1) $$Y(s)={\tilde{\beta }}_{0}+\beta_{1}\tilde{x}(s)+\varepsilon (s)$$

where Y(s) is the observed concentration at point s, $\tilde{x}$(s) is the CMAQ concentration at point s based on the weighted average of both the grid cell containing the monitor and neighboring grid cells (in our application, up to three neighboring grid cells were used in each direction from the monitor location *s*, with the grid weights decreasing exponentially over distance as described above), ${\tilde{\beta }}_{0}$(s) is the intercept where ${\tilde{\beta }}_{0}(s)=\beta_{0}+\beta_{0}(s)$ is composed of both a global component, β_0_, and a local component, β_0_(s), that is modelled as a mean-zero Gaussian process with exponential decay, β_1_ is the global slope, and *ε*(s) is the model error. A comprehensive discussion of the DS methodology is given in Heaton et al. [13]. This framework allows for multiple levels of spatial dependence, where the spatially varying regression coefficient describes the spatial relationship between monitoring stations, with dependence decaying over the distance between sites, and the spatial dependence between CMAQ grid cells is accounted for via the spatially varying weighted derived regressors. Conjugate priors are used for the spatial covariates and the error variance, with the spatial decay parameters estimated via a grid search placing discrete mass at varying percentages of the maximum observed differences from the monitor location (as described in Heaton et al. [13]). The DS modeling framework uses Markov chain Monte Carlo methods to draw random samples from iteratively updated parameter distributions during the parameter estimation, allowing for uncertainty quantification for the resulting predictions via estimates of the standard error associated with these sampling distributions. The Downscaler model is fully described in a series of three papers [9,14,15], with the version of DS used here based on Berrocal et al. [9].

In this work, we evaluated the impact of adding Interagency Monitoring of Protected Visual Environments (IMPROVE) PM_2.5_ concentrations from sites in remote regions to the DS inputs. Typical DS applications use only PM_2.5_ measurements from AQS sites, whose land use is heavily urban in nature (i.e., biased towards urban). Specifically, we conducted DS model simulations with and without IMPROVE measurements to quantify the scale and direction of the changes in DS PM_2.5_ predictions. Two annual DS model runs were performed in this study to predict PM_2.5_ concentrations at a 12 × 12 km^2^ grid resolution for the year 2014 using the CMAQv5.1 continental U.S. domain. For both model runs, a consistent set of monitoring (2014 ambient PM_2.5_ concentrations at the urban-focused AQS sites) and modeling (CMAQ predictions from a 2014 annual simulation) inputs were used, with the second run also incorporating data from the IMPROVE network. These IMPROVE PM_2.5_ concentration data were added without modifications to test the impact on the DS predictions. This evaluation not only quantifies the impact of the new monitoring inputs to DS but also identifies the domain-wide effect of adding a geographically distinct (in this case only remote sites) observational dataset to DS. The previous DS model runs performed by the EPA for the NAAQS and CDC used only regulatory-quality PM_2.5_ concentrations from the AQS network as inputs, and therefore did not include the IMPROVE PM_2.5_ data.

## 3. Data Sources

**Figure 1.**
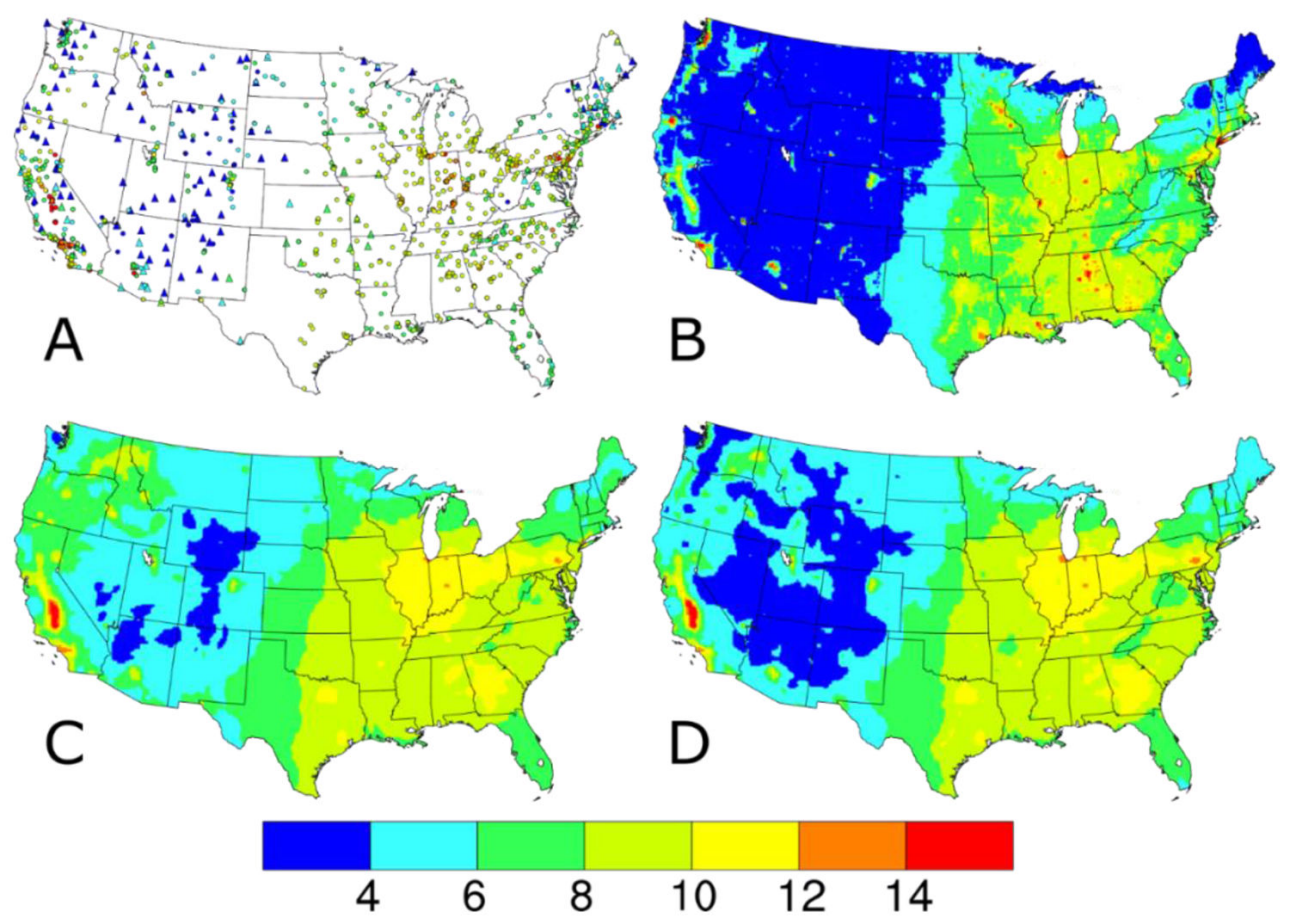
Annual average PM_2.5_ concentrations (in units of μg m^−3^) (**A**) observed at AQS (circles) and IMPROVE (triangles) sites and predicted on IMPROVE sample dates by the (**B**) CMAQ model, (**C**) Downscaler with the baseline configuration, and (**D**) Downscaler with IMPROVE data.

**Figure 2.**
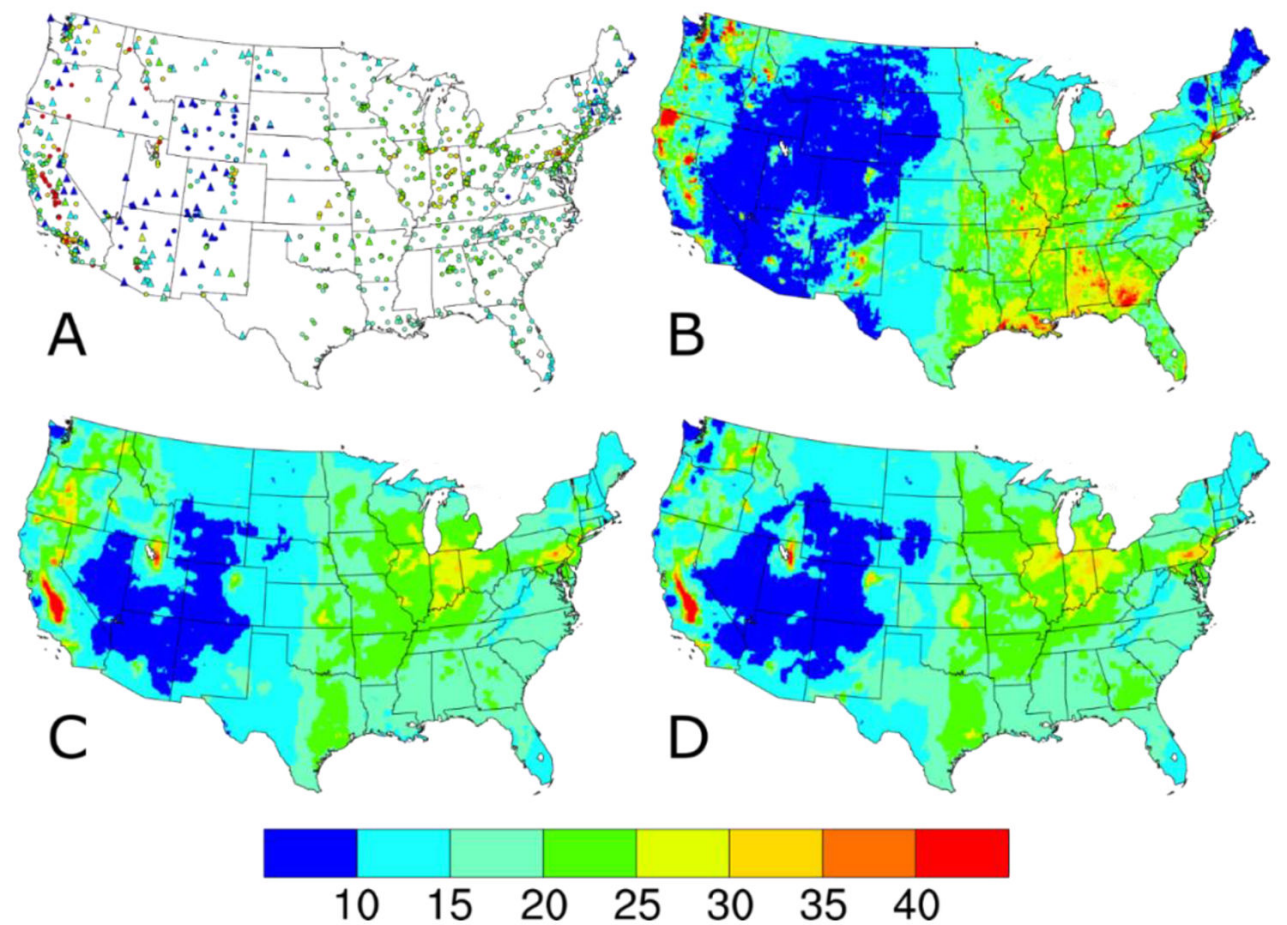
98th percentile PM_2.5_ concentrations (in units of μg m^−3^) (**A**) observed at AQS (circles) and IMPROVE (triangles) sites and predicted on IMPROVE sample dates by the (**B**) CMAQ model, (**C**) Downscaler with the baseline configuration, and (**D**) Downscaler with IMPROVE data.

The AQS PM_2.5_ concentrations include only the 24-h average regulatory-quality measurements from the federal reference method (FRM) or federal equivalent method (FEM) monitors across the 48 contiguous United States. Monitors at AQS sites measure regulatory-quality PM_2.5_ concentrations on a variety of sampling frequencies, with roughly 13%, 47%, and 40% of sites having monitors operating on a 1-in-6 day, 1-in-3 day, or daily sampling, respectively, in 2014. No annual completeness criteria were applied to the AQS sites, but missing daily data were ignored in the DS model runs. The AQS input datasets were summarized by annual means (circles, Figure 1A) and 98th percentile values (circles, Figure 2A) to approximate the averaging times of the PM_2.5_ NAAQS. The 934 AQS sites, shown as the circles in Figures 1A and 2A, are relatively widespread in the eastern U.S. and California and sparse in the Intermountain West. This spatial distribution is a function of the network design intent, which focuses on higher concentration areas and populated areas.

In 2014, the IMPROVE network [16,17] consisted of 153 sites located in remote areas, like National Parks and Wilderness Areas mostly in the western U.S. (hereafter defined as the region with a longitude west of 100° W containing 98 IMPROVE sites). IMPROVE sites measure concentrations of PM_2.5_ mass and concentrations of PM_2.5_ chemical components on the same 1-in-3-day schedule throughout the U.S. Although PM_2.5_ mass concentrations are measured at IMPROVE sites using a filter-based method similar to an FRM, it is not considered regulatory quality [17]. The IMPROVE input datasets were summarized by annual means (triangles, Figure 1A) and 98th percentile values (triangles, Figure 2A). The IMPROVE sites fill in the spatial gaps in many of these Intermountain West areas.

Of the 153 IMPROVE sites, 17 were collocated with AQS sites and can be intercompared. Analysis of the 2014 PM_2.5_ observations from temporally and spatially collocated IMPROVE and AQS measurements indicates that the IMPROVE annual averages are biased low (−15%) compared to AQS annual averages but with a difference that is not statistically significant (*p*-value = 0.368). This bias is similar to the results of Hand et al. [18], which compared monthly averages from six collocated IMPROVE and CSN sites between 2008 and 2011. We also found that 2014 IMPROVE and AQS PM_2.5_ data at the 17 collocated sites have high correlation coefficients (0.72 to 0.99) and slopes near unity (0.73 to 1.13). Only six IMPROVE sites in the western U.S. are collocated with AQS sites; analysis of the 2014 PM_2.5_ observations from temporally and spatially collocated IMPROVE and AQS measurements at these western U.S. sites indicates that the low bias of the IMPROVE annual averages is reduced (−7%) and also not statistically significant.

The 2014 CMAQ annual simulation used version 5.1 coupled to the Weather Research and Forecasting (WRF) version 3.7.1 for the meteorological inputs [19,20]. The CMAQ model configuration included bi-directional ammonia (NH_3_) air-surface exchange (v2.1) using the Massad formulation [21], CB05e51 chemical mechanism, AERO6 aerosol module, and lightning NO_x_-adjusted to lightning strike data. The PM_2.5_ concentrations predicted by CMAQ were calculated by adding up the concentrations of several chemical species including sulfate, nitrate, ammonium, sodium, chloride, elemental carbon, organic matter, and other minor components and multiplying those values by the fraction of the three model modes with diameters <2.5 μm. The CMAQ input datasets were summarized by annual averages (Figure 1B) and 98th percentiles (Figure 2B). Note the complete spatial coverage and qualitative similarity in the national spatial gradients seen in the AQS and IMPROVE measurements.

In order to study the impact of the inclusion of the IMPROVE data on DS predictions, the two DS model runs consisted of (1) a “baseline” run using AQS inputs without IMPROVE data and (2) a “w/IMPROVE” run, which includes both AQS and IMPROVE data, i.e., the inclusion of IMPROVE data being the only difference between the two model runs. For both model runs, every day of 2014 was simulated despite the fact that all IMPROVE sites operate on the same 1-in-3-day schedule and only affect those days in the DS model runs. Therefore, we report the DS results only for days with IMPROVE sample (hereafter “IMPROVE sample dates”) in the main text but show the results for all days in 2014 (hereafter “entire period”) in the supplemental information to provide a context for the impact on a typical application of the DS model. In the following section, we compare the annual average and 98th percentile values predicted by the two DS model runs as well as evaluate DS predictions to the AQS and IMPROVE PM_2.5_ data used as in the input to the model.

## 4. Results

### 4.1. Concentrations from Downscaler Model Runs

Compared to the CMAQ-predicted PM_2.5_ annual average and 98th percentile concentrations in Figures 1B and 2B, respectively, the annual average and 98th percentile PM_2.5_ values from all DS model runs (Figure 1C,D and Figure 2C,D, respectively) have smoother gradients and fewer areas at the extremes of the data range. This can be seen for the lower values in Maine, and in Georgia and Alabama as well as Louisiana for the higher values, with differences in both lower and higher values shown in Washington. The differences between the baseline and w/IMPROVE DS annual average predictions are largest in areas near IMPROVE monitors across the western U.S., the Ozarks region of Arkansas, and the Appalachian Mountains. For the 98th percentile DS predictions, a similar pattern of smoother gradients than CMAQ and less extreme values emerges for both the baseline and w/IMPROVE DS model runs. For the entire period (see Figures S1 and S2), the CMAQ and baseline DS annual average and 98th percentile values are similar to those on IMPROVE sample dates.

### 4.2. Differences in Concentrations between Downscaler Model Runs

**Figure 3.**
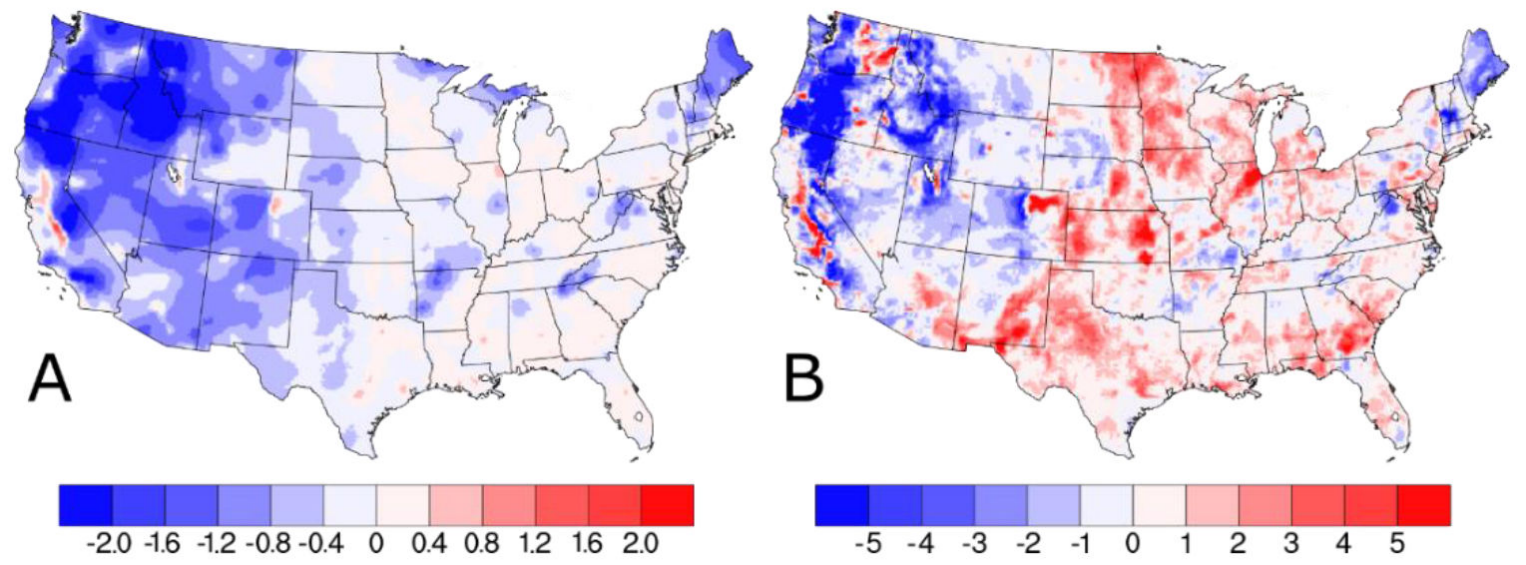
Difference in (**A**) annual average and (**B**) 98th percentile PM_2.5_ concentrations (in units of μg m^−3^) on IMPROVE sample dates as predicted by Downscaler when the IMPROVE data are included.

The w/IMPROVE—baseline difference plot in Figure 3A showing the difference in annual average w/IMPROVE DS and baseline predictions identifies three distinct regions: (1) Isolated areas in the San Joaquin Valley, CA and Denver, CO with increased PM_2.5_ (>0.5 μg m^−3^), (2) widespread areas in the central and eastern U.S. with little change or slight increases (0–0.4 μg m^−3^), and (3) widespread areas in the western U.S. where PM_2.5_ predictions were reduced by more than 1.0 μg m^−3^. The largest reduction in PM_2.5_ predictions were in states like Idaho and Oregon, where a smaller number of AQS sites with moderate PM_2.5_ values are combined with several IMPROVE sites with very low PM_2.5_ to reduce PM_2.5_ predictions by more than 2.0 μg m^−3^. Isolated areas, such as San Joaquin Valley, CA and Denver, CO, with increased PM_2.5_ concentrations in the w/IMPROVE DS model run are near high elevation areas with reduced PM_2.5_; this results in a sharper gradient of PM_2.5_ concentrations in these regions predicted by the w/IMPROVE DS model run. This is likely due to a decrease in spatial covariance, allowing for both higher/lower local concentrations. Relative to the annual average difference plot, the 98th percentile difference plot for w/IMPROVE—baseline in Figure 3B shows fewer areas with reduced PM_2.5_ concentrations (mainly in Oregon) and more areas with slightly higher PM_2.5_ (mainly in the central U.S.). For the entire period, these annual and 98th percentile differences are smaller (see Figure S3).

**Figure 4.**
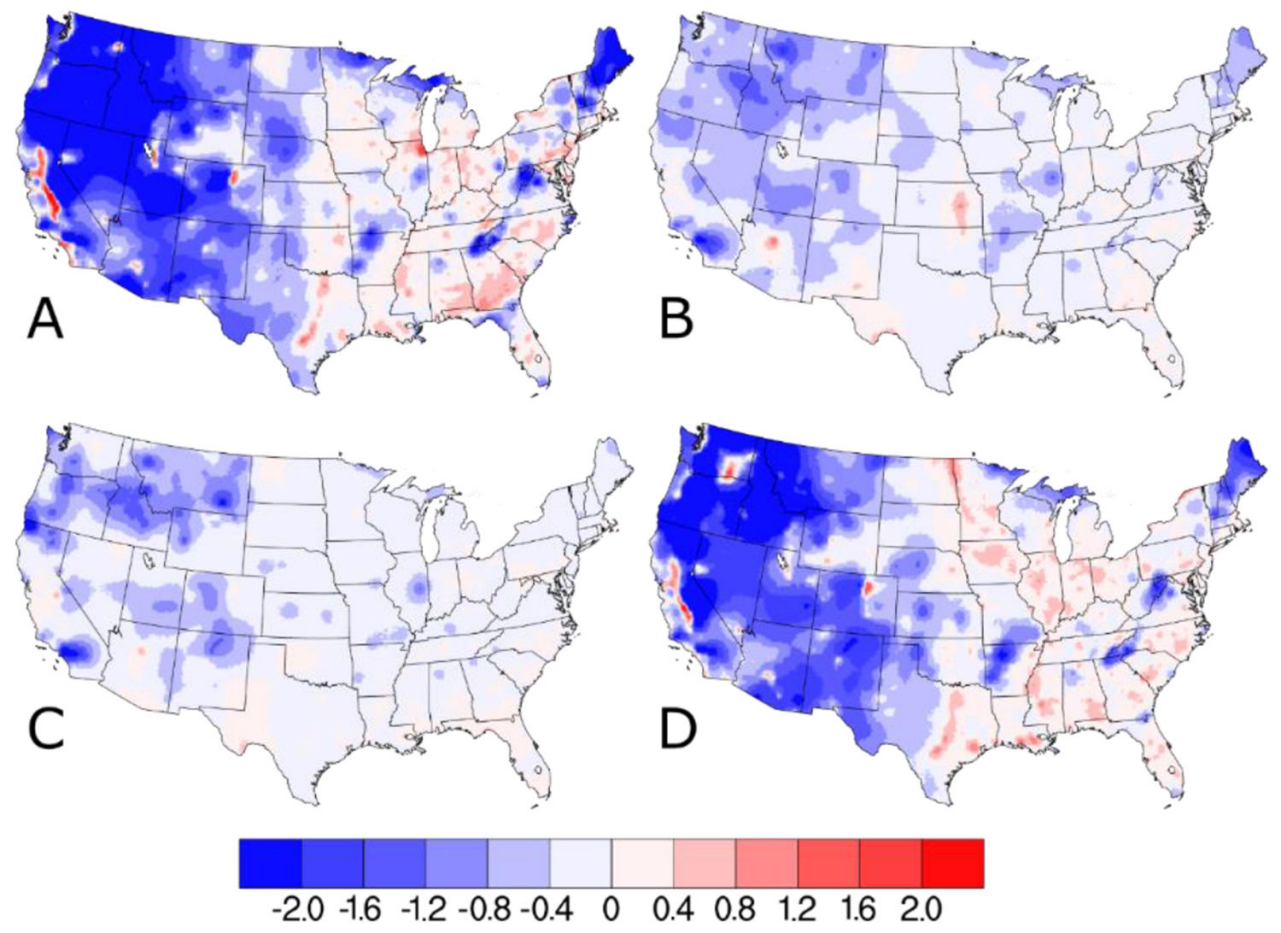
Difference in (**A**) Q1 (January, February, March), (**B**) Q2 (April, May, June), (**C**) Q3 (July. August, September), and (**D**) Q4 (October, November, December) quarterly average PM_2.5_ concentrations (in units of μg m^−3^) on IMPROVE sample dates as predicted by Downscaler when the IMPROVE data are included.

When the differences between the w/IMPROVE and baseline DS predictions are separated into quarterly averages, a strong seasonal cycle is evident (see Figure 4 and Figure S4). Relative to the warmer months in quarters 2 and 3, the colder months of quarter 1 and quarter 4 have much larger changes in the w/IMPROVE DS predictions (both increases and decreases). The cause of this seasonality is as follows: (1) The absolute wintertime PM_2.5_ concentrations are higher than in other seasons for most areas of the U.S. and (2) wintertime inversions contribute to much higher PM_2.5_ concentrations in the valleys (where AQS sites are typically located) than at higher elevations (where IMPROVE sites are typically located), and (3) summertime PM_2.5_ concentrations in most areas of the U.S. are dominated by secondary components, such as sulfate and organic carbon, which affect both urban and remote areas [22]. By virtue of the 1-in-3-day sampling schedule for all the IMPROVE sites across the U.S., 2/3 of the days have no impact from the IMPROVE data and are identical to the baseline DS model run. Figures S1 and S2 indicate that the impact of including the IMPROVE PM_2.5_ data in DS is much smaller for the entire period. The DS modeling framework also generates standard error estimates, which quantify the model uncertainty. The difference plot of the standard error estimates for the w/IMPROVE and baseline model runs (Figure S5a) shows that there is a general trend of much lower errors over much of the western U.S. and moderately higher errors over much of the eastern U.S. Near the IMPROVE sites, the w/IMPROVE predictions consistently have lower errors regardless of geography. Much like the predicted concentration differences, the standard error differences between the two DS model runs are smaller for the entire period (see Figure S5b).

### 4.3. Downscaler Evaluation

**Figure 5.**
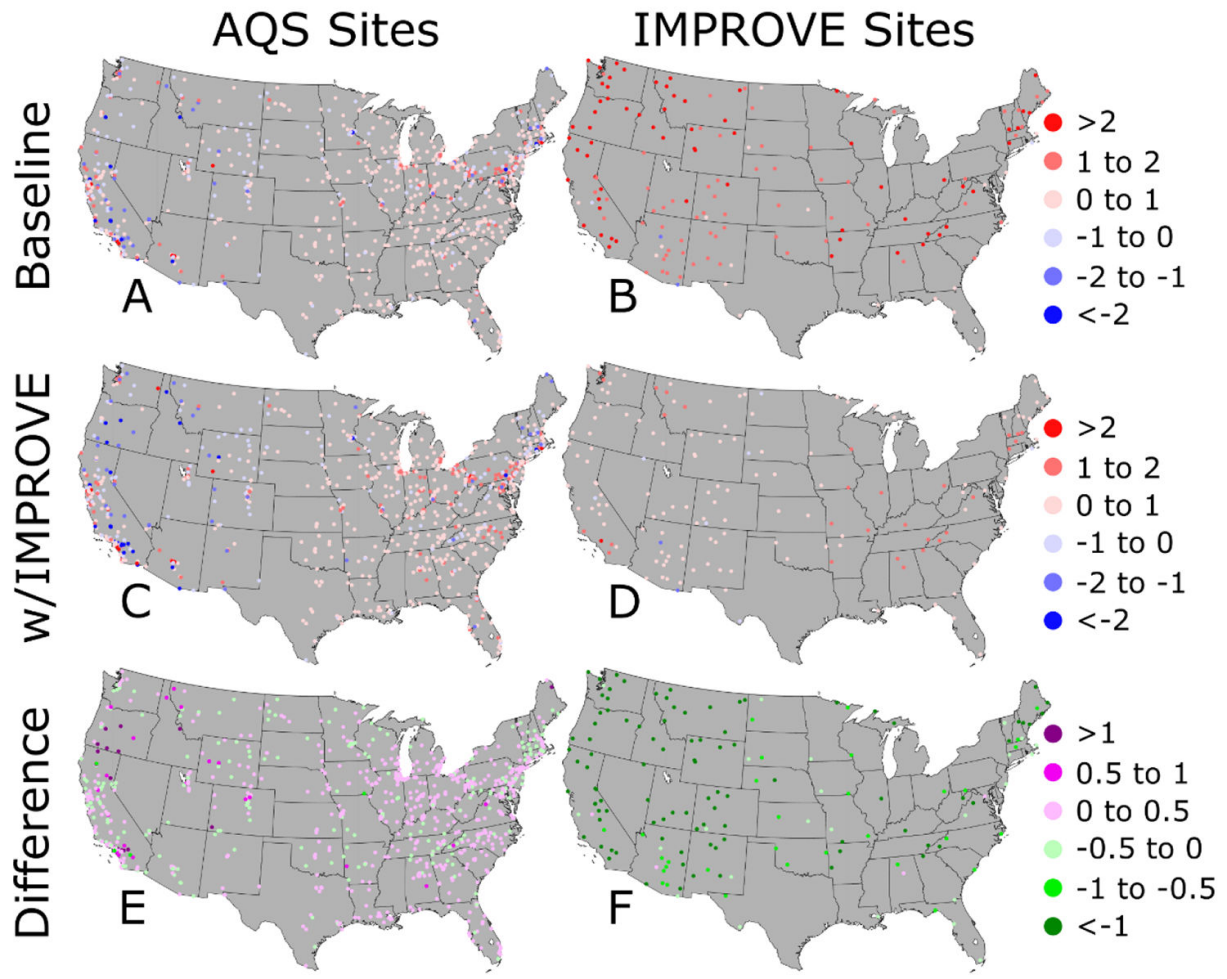
Mean bias for the (**A,B**) baseline Downscaler prediction, (**C,D**) Downscaler prediction with IMPROVE data, and the difference in the absolute mean bias between the two model runs for the PM_2.5_ observations at (**E**) AQS and (**F**) IMPROVE sites for 2014. The green colors on the bottom row figures represent locations where the Downscaler predictions with IMPROVE data had a lower model bias (improved prediction) and purple colors represent locations where the Downscaler predictions with IMPROVE data had a higher model bias (worse prediction).

**Figure 6.**
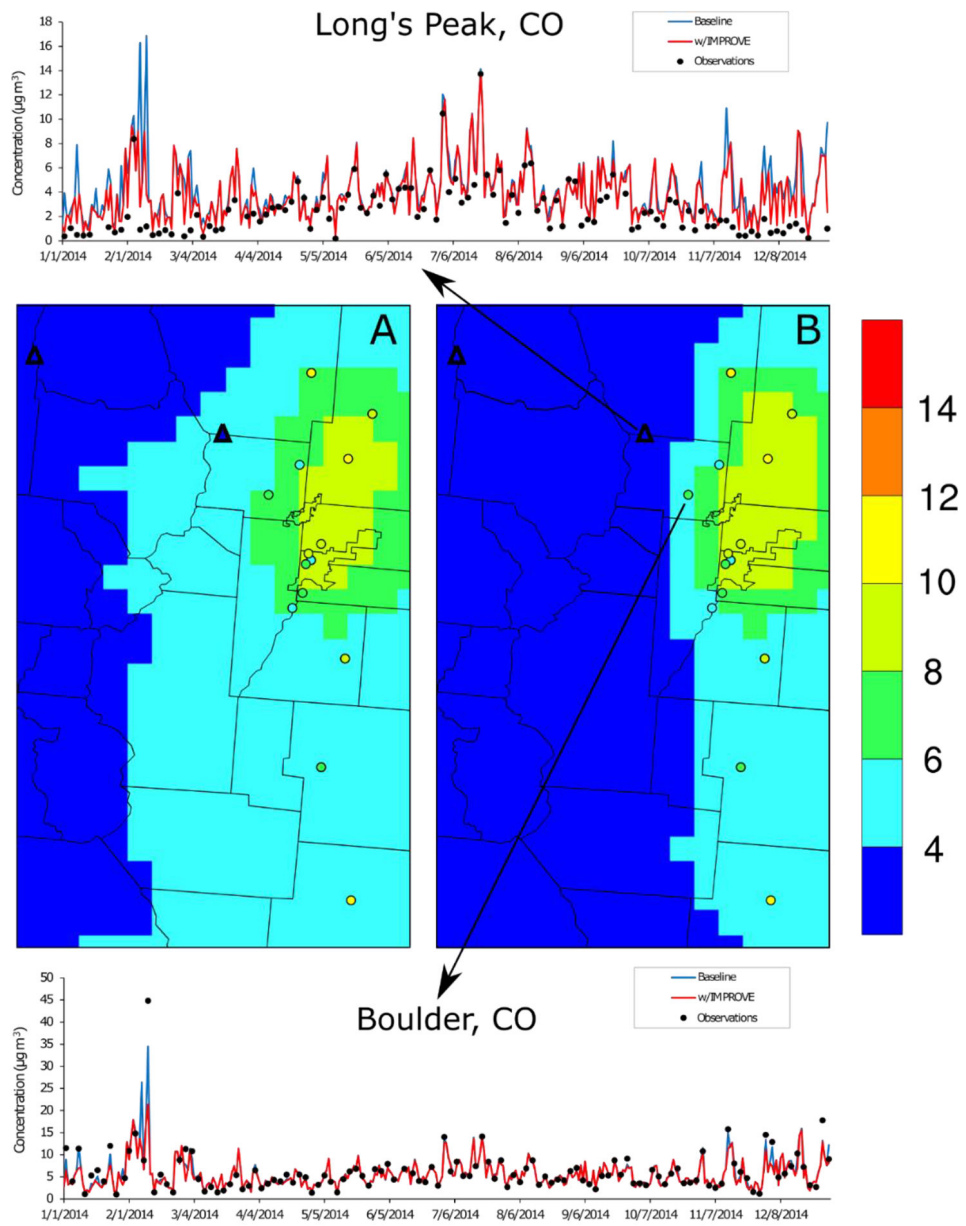
Denver, Colorado-area annual average PM_2.5_ concentrations on IMPROVE sample dates that were (**A**) predicted by Downscaler with the baseline configuration and (**B**) predicted by Downscaler with IMPROVE data superimposed with annual averages observed at the AQS (circles) and IMPROVE (triangles) sites. The time series charts give the 24-h average PM_2.5_ concentrations observed and predicted at Long’s Peak and Boulder, CO.

If we assume that the DS prediction at the grid centroid is consistent with the grid average, the PM_2.5_ concentrations at the AQS and IMPROVE sites can be used to evaluate the baseline and w/IMPROVE DS model runs. For the AQS sites, Figure 5 shows that the PM_2.5_ concentrations are well-predicted (mean bias within ±1 μg m^−3^) by both the baseline (Figure 5A) and w/IMPROVE (Figure 5C) DS model runs; this result is not surprising because these data are used as input in both. The absolute mean bias difference (∣w/IMPROVE – AQS∣ – ∣baseline – AQS∣) plot in Figure 5E identifies the location of AQS sites with improved or degraded predictions by the w/IMPROVE DS model run relative to the baseline. Most of these sites had slight increases or decreases in their mean bias, but a few had their mean biases degrade by >0.5 μg m^−3^. The AQS sites with degraded predictions in the w/IMPROVE DS model run were clustered in areas near multiple IMPROVE sites, including parts of California and Oregon, with underpredictions that were made worse by introducing low PM_2.5_ concentrations from the IMPROVE sites (Figure 6).

For the IMPROVE sites, Figure 5B shows that the baseline DS model run almost universally overpredicts concentrations with many sites having a mean bias >2 μg m^−3^. Overpredictions at the IMPROVE sites are also common in the w/IMPROVE DS model run (Figure 5D), but most sites have a mean bias within ±1 μg m^−3^. The absolute mean bias difference in Figure 5F shows that nearly all predictions in the w/IMPROVE DS model run were better than that of the baseline, often by more than 1 μg m^−3^. Of the few IMPROVE sites with degraded predictions in the w/IMPROVE DS model run, most were clustered near AQS sites whose concentrations are underpredicted in both DS model runs.

Domain-wide validation statistics (based on sensitivity runs where 10% of the input data is left out) were also calculated for the baseline and w/IMPROVE model runs, which we limited only to (1) AQS sites and (2) the common site-days (1889 or ~10% of withheld baseline DS site-days) from the two model runs. When looking at withheld AQS site-days, the w/IMPROVE bias (0.18 μg m^−3^) is significantly lower than baseline (0.29 μg m^−3^) while the mean squared error and fraction of time that the prediction is included within the 95% confidence interval (aka, coverage) are not significantly different. When looking at the common site-days withheld in both simulations, the w/IMPROVE model has a lower bias (0.30 μg m^−3^) than baseline (0.35 μg m^−3^) but the low number of site-days led to no performance differences being statistically significant. Random withholding can in some cases underestimate actual model error [23]. In this case, the error is being used in a relative sense to compare two simulations, so the effect is not expected to be influential. More details of the validations are available in Table 1. Scatter density plots of the validation results for the baseline and w/IMPROVE DS model runs when AQS site-days are withheld (Figure S6) show that the correlation coefficients are similar between the two model runs and are comparable to those reported in a cross-validation experiment by Berrocal et al. [24].

### 4.4. Case Study for Denver, CO

An illustrative example of DS predictions near Denver, Colorado (Figure 6) gives some insight to the impact of including IMPROVE data in the DS model run. In the Denver region, an urban core with high PM_2.5_ concentrations monitored by AQS sites is near high elevation areas with low PM_2.5_ concentrations monitored by IMPROVE sites. The numerous AQS and IMPROVE sites near Denver, CO combine to inform the gradients in the DS-predicted PM_2.5_ between the urban core and nearby mountains. The gradients are sharper in the w/IMPROVE DS model run, and while the IMPROVE site nearest to the urban core (Long’s Peak) is overpredicted in both DS model runs the overprediction is mitigated in the w/IMPROVE DS model. This is seen in both the annual average spatial field as well as across the 2014 time series (Figure 6). DS performance at the AQS site on the edge of the urban core (Boulder, CO, USA) has the opposite problem; in the baseline model run, the underpredictions are increased under the w/IMPROVE run. This underprediction is especially evident on high PM_2.5_ concentration days at Boulder, CO when the w/IMPROVE DS model run is trying to fuse these data with much lower PM_2.5_ concentrations at the nearby IMPROVE site. The time series plots for the IMPROVE site in Figure 6 also shows an unintended consequence of using the IMPROVE data as input to DS; because all IMPROVE sites sample on the same schedule (1-in-3 days) throughout the United States, the w/IMPROVE DS model run has more day-to-day variation than the baseline run for the entire period.

## 5. Conclusions

Prior to completing the DS model runs, we expected that incorporating IMPROVE data would result in better predictions at both AQS and IMPROVE sites compared to the baseline using only AQS data. The results were more nuanced. The IMPROVE sites were much better predicted by the inclusion of IMPROVE data, but some of the AQS sites experienced a slight degradation in predictions. The effect of including IMPROVE data is most obvious in preserving broad low-concentration areas in the western U.S. that are present in both the CMAQ model and the IMPROVE network but are absent in the AQS-only DS predictions. In areas like Denver, CO with strong gradients of PM_2.5_ concentrations associated with elevation changes, incorporating IMPROVE data in DS enhances those gradients. These examples highlight the effect of a bias in the AQS network towards urban and polluted areas that impacts DS predictions at unmonitored and/or more rural locations. Incorporating the relatively low PM_2.5_ concentrations from IMPROVE sites to the DS model also resulted in an increase in predicted concentrations for some areas. Overall, the cross-validation results of common site-days withheld in both simulations show both a slight reduction in the mean bias and a slight increase in the mean square error when the IMPROVE data is included. These nonintuitive results are due to the combination of global and local components of the DS equations and the pollutant process itself, suggesting that without the IMPROVE data the spatial covariance in the baseline DS model run is overestimated. They suggest that the domain (national- or urban-focused) and the type of data inputs to DS must be carefully considered.

Another consideration for the incorporation of the IMPROVE network data is the day-to-day variation in the PM_2.5_ predictions, particularly near IMPROVE sites. The effect of leaving out IMPROVE observations can only be evaluated for 1-in-3 days but is implicitly present on all days. When performing daily DS simulations, only 1/3 of all days are affected by IMPROVE and much of the impacts are lost (see Figures S1 and S2). Even with IMPROVE 1-in-3-day observations, the average day observational input is still biased towards urban and polluted areas. This highlights the need to either incorporate other sources of data with greater temporal coverage (e.g., AOD derived from a geostationary satellite like the Geostationary Operational Environmental Satellite (GEOS) series) and/or higher spatial resolution (e.g., 4 km CMAQ domain), or to apply DS using time aggregates (e.g., monthly average) to minimize the unmonitored time periods. The use of IMPROVE and/or other irregular inputs to data fusion techniques need to be carefully considered when utilizing DS predictions. This study does not address the ability of CMAQ or other potential DS inputs to replicate the PM_2.5_ surface; using high-quality inputs is fundamental for any data fusion application regardless of the methodology.

The distinct change in DS model bias at IMPROVE vs. AQS sites shows that more input data for fusion applications does not always directly improve all predictions. The consistent reduction in bias when adding IMPROVE measurements suggests that the baseline DS increases predictions in rural areas as the original inputs are based on urban monitors. The worsening underpredictions at AQS sites when adding IMPROVE to DS suggests that concentrations at urban sites are reduced by addition of information from generally lower concentration rural monitors. This may point to a need to differentiate PM_2.5_ species with long lifetimes (i.e., secondary species) and short lifetimes (i.e., primary species) when applying the statistical downscaling approaches. More research is needed to further constrain appropriate scales of influence.

## Supplementary Material

SI

## Figures and Tables

**Table 1.** Validation (10% of site-days randomly withheld) results for 2014 Downscaler model runs (baseline and w/IMPROVE) for all site-days (All), AQS site-days (AQS), or all common site-days (common). The statistics shown include the number of site-days (N), mean bias, mean square error, and coverage. Here, 90% confidence intervals are shown in parentheses.

Simulation	Selection	N	Mean Bias	Mean Sq. Error	Coverage
Baseline	AQS	18,518	0.29 (0.25, 0.33)	10.85 (9.86, 11.84)	0.95 (0.95, 0.96)
w/IMPROVE	All	20,257	0.28 (0.24, 0.32)	11.51 (10.43, 12.60)	0.95 (0.94, 0.95)
w/IMPROVE	AQS	17,317	0.18 (0.14, 0.23)	11.87 (10.65, 13.09)	0.95 (0.95, 0.95)
Baseline	Common	1889	0.35 (0.24, 0.46)	8.67 (7.42, 9.92)	0.95 (0.94, 0.96)
w/IMPROVE	Common	1889	0.30 (0.19, 0.41)	8.69 (7.41, 9.98)	0.96 (0.95, 0.97)

## References

[1] Crouse DL; Peters PA; Hystad P; Brook JR; van Donkelaar A; Martin RV; Villeneuve PJ; Jerrett M; Goldberg MS; Pope CA; Ambient PM_2.5_, O_3_, and NO_2_ Exposures and Associations with Mortality over 16 Years of Follow-Up in the Canadian Census Health and Environment Cohort (CanCHEC). Environ. Health Perspect 2015, 123, 1180–1186. 26528712 10.1289/ehp.1409276 PMC4629747

[2] Shi LH; Zanobetti A; Kloog I; Coull BA; Koutrakis P; Melly SJ; Schwartz JD Low-Concentration PM_2.5_ and Mortality: Estimating Acute and Chronic Effects in a Population-Based Study. Environ. Health Perspect 2016, 124, 46–52. 26038801 10.1289/ehp.1409111 PMC4710600

[3] Di Q; Kloog I; Koutrakis P; Lyapustin A; Wang YJ; Schwartz J Assessing PM_2.5_ Exposures with High Spatiotemporal Resolution across the Continental United States. Environ. Sci. Technol 2016, 50,4712–4721. 27023334 10.1021/acs.est.5b06121 PMC5761665

[4] Liu Y; Sarnat J; Kilaru V; Jacob D; Koutrakis P Estimating ground-level PM_2.5_ in the eastern United States using satellite remote sensing. Environ. Sci. Technol 2005, 39, 3269–3278. 15926578 10.1021/es049352m

[5] Liu Y; Paciorek CJ; Koutrakis P Estimating regional spatial and temporal variability of PM_2.5_ concentrations using satellite data, meteorology, and land use information. Environ. Health Perspect 2009, 117, 886–892. 19590678 10.1289/ehp.0800123 PMC2702401

[6] Gupta P; Christopher SA Particulate matter air quality assessment using integrated surface, satellite, and meteorological products: Multiple regression approach. J. Geophys. Res 2009, 114, D14205.

[7] Van Donkelaar A; Martin RV; Brauer M; Hsu NC; Kahn RA; Levy RC; Lyapustin A; Sayer AM; Winker DM Global estimates of fine particulate matter using a combined geophysical-statistical method with information from satellites, models, and monitors. Environ. Sci. Technol 2016, 50, 3762–3772. 26953851 10.1021/acs.est.5b05833

[8] Shaddick G; Thomas ML; Green A; Brauer M; Van Donkelaar A; Burnett R; Chang HH; Cohen A; Van Dingenen R; Dora C; Data integration model for air quality: A hierarchical approach to the global estimation of exposures to ambient air pollution. J. R. Stat. Soc. Ser. C (Appl. Stat.) 2018, 67, 231–253.

[9] Berrocal V; Gelfand AE; Holland DM Space-time fusion under error in computer model output: An application to modeling air quality. Biometrics 2012, 68, 837–848. 22211949 10.1111/j.1541-0420.2011.01725.x PMC4442701

[10] Policy Assessment for the Review of the National Ambient Air Quality Standards for Particulate Matter, External Review Draft, Environmental Protection Agency. Available online: https://www.epa.gov/naaqs/particulate-matter-pm-air-quality-standards (accessed on 26 December 2019).

[11] National Environmental Public Health Tracking Network, Centers for Disease Control and Prevention. Available online: https://www.cdc.gov/nceh/tracking/default.htm (accessed on 26 December 2019).

[12] Byun DW; Schere KL Review of the Governing Equations, Computational Algorithms, and Other Components of the Models-3 Community Multiscale Air Quality (CMAQ) Modeling System. Appl. Mech. Rev 2006, 59, 51–77.

[13] Heaton M; Holland DM; Leininger T User’s Manual for Downscaler Fusion Software; EPA/600/C-12/002; U.S. Environmental Protection Agency: Washington, DC, USA, 2012.

[14] Berrocal V; Gelfand AE; Holland DM A spatiotemporal downscaler for output from numerical models. J. Agric. Biol. Environ. Stat 2010, 15, 176–197. 21113385 10.1007/s13253-009-0004-z PMC2990198

[15] Berrocal V; Gelfand AE; Holland DM A bivariate space-time downscaler under space and time misalignment. Ann. Appl. Stat. 2010, 4, 1942–1975. 21853015 10.1214/10-aoas351 PMC3156619

[16] Malm WC; Sisler JF; Huffman D; Eldred RA; Cahill TA Spatial and seasonal trends in particle concentration and optical extinction in the U.S. J. Geophys. Res 1994, 99, 1347–1370.

[17] Solomon PA; Crumpler D; Flanagan JB; Jayanty RKM; Rickman EE; McDade CE U.S.national PM_2.5_ chemical speciation monitoring networks-CSN and IMPROVE: Description of networks. J. Air Waste Manag. Assoc 2014, 64, 1410–1438. 25562937 10.1080/10962247.2014.956904

[18] Hand JL; Schichtel BA; Malm WC; Pitchford M; Frank NH Spatial and seasonal patterns in urban influence on regional concentrations of speciated aerosols across the United States. J. Geophys. Res 2014, 119, 12832–12849.

[19] Appel KW; Napelenok SL; Foley KM; Pye HOT; Hogrefe C; Luecken DJ; Bash JO; Roselle SJ; Pleim JE; Foroutan H; Description and evaluation of the Community Multiscale Air Quality (CMAQ) modeling system version 5.1. Geosci. Model Dev 2017, 10, 1703–1732. 30147852 10.5194/gmd-10-1703-2017 PMC6104654

[20] Skamarock WC; Klemp JB A time-split nonhydrostatic atmospheric model for weather research and forecasting applications. J. Comput. Phys 2008, 227, 3465–3485.

[21] Massad RS; Nemitz E; Sutton MA Review and parameterization of bi-directional ammonia exchange between vegetation and the atmosphere. Atmos. Chem. Phys 2010, 10, 10359–10386.

[22] Chan EAW; Gantt B; McDow S The reduction of summer sulfate and switch from summertime to wintertime PM_2.5_ concentration maxima in the United States. Atmos. Environ 2018, 175, 25–32. 10.1016/j.atmosenv.2017.11.055 PMC6134864 30220859

[23] Roberts DR; Bahn V; Ciuti S; Boyce MS; Elith J; Guillera-Arroita G; Hauenstein S; Lahoz-Monfort JJ; Schröder B; Thuiller W; Cross-validation strategies for data with temporal, spatial, hierarchical, or phylogenetic structure. Ecography 2017, 40, 913–929.

[24] Berrocal VJ; Guan Y; Muyskens A; Wang H; Reich BJ; Mulholland JA; Chang HH A comparison of statistical and machine learning methods for creating national daily maps of ambient PM_2.5_ concentration. Atmos. Environ 2019, in press. 10.1016/j.atmosenv.2019.117130 PMC7451200 32863727

