# Supplementary material for: Incorporation of Remote PM2.5 Concentrations into the Downscaler Model for Spatially Fused Air Quality Surfaces
Source: Atmosphere (Basel). Author manuscript; Available in PMC 2020 Jul 7. (PMC7339729; doi:10.3390/atmos11010103)
Supplement: SI [file NIHMS1575092-supplement-SI.pdf]

Supporting Information

# Incorporation of remote $PM_{2.5}$ concentrations into the Downscaler Model for spatially-fused air quality surfaces

Brett Gantt[1], Kelsey McDonald[2], Barron Henderson[1], Elizabeth Mannshardt[1]

[1]Office of Air Quality Planning and Standards, Environmental Protection Agency, Research Triangle Park, 27711, USA
[2]Department of Psychology & Neuroscience, Duke University, Durham, 27708, USA

*Correspondence to*: Elizabeth Mannshardt (mannshardt.elizabeth@epa.gov) or Brett Gantt (gantt.brett@epa.gov)

Contains 6 figures

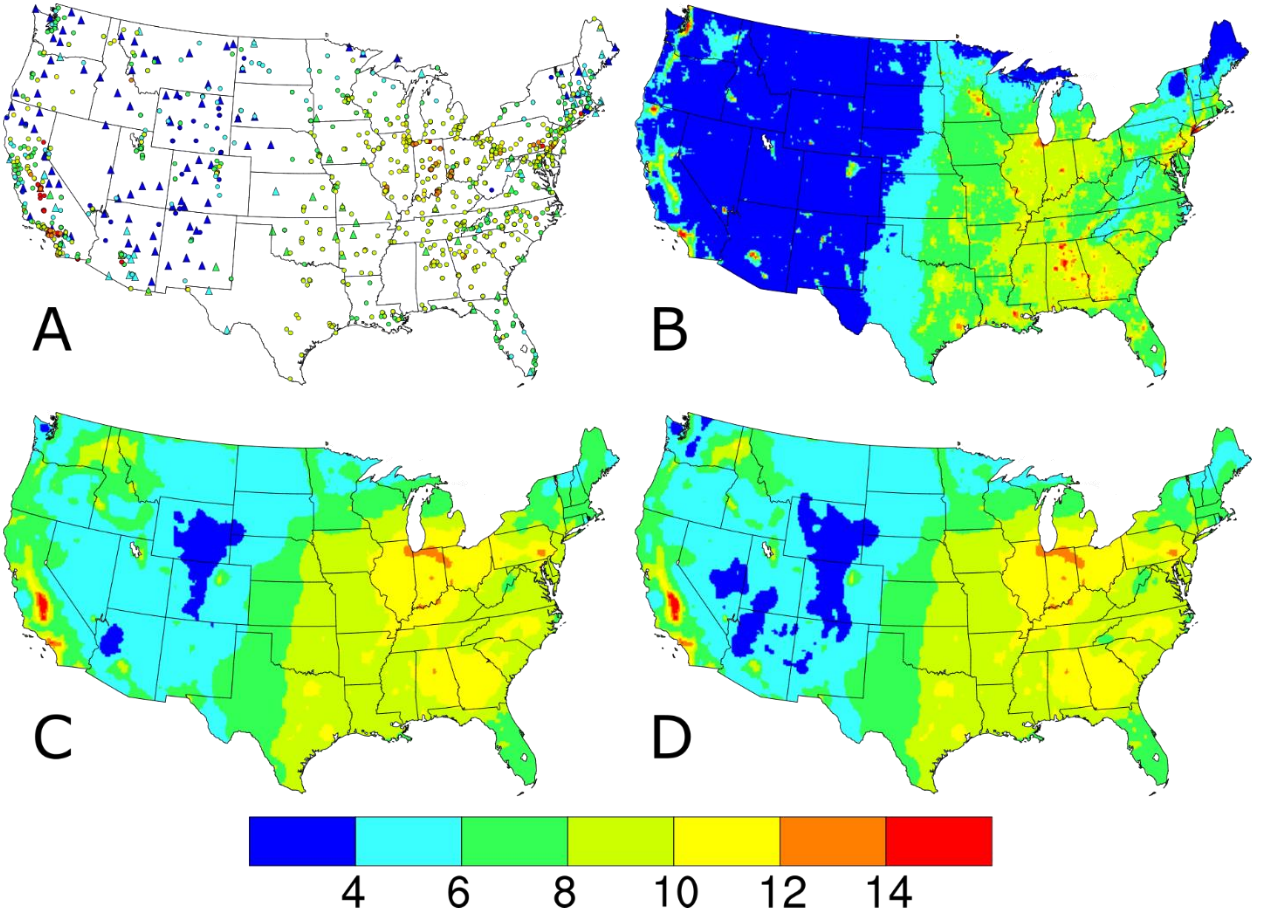


**Figure S1: Annual average $PM_{2.5}$ concentrations (in units of $\mu g\ m^{-3}$) a) observed at the AQS (circles) and IMPROVE (triangles) sites and predicted for the entire period by the b) CMAQ model, c) Downscaler with the baseline configuration, and d) Downscaler with IMPROVE data.**

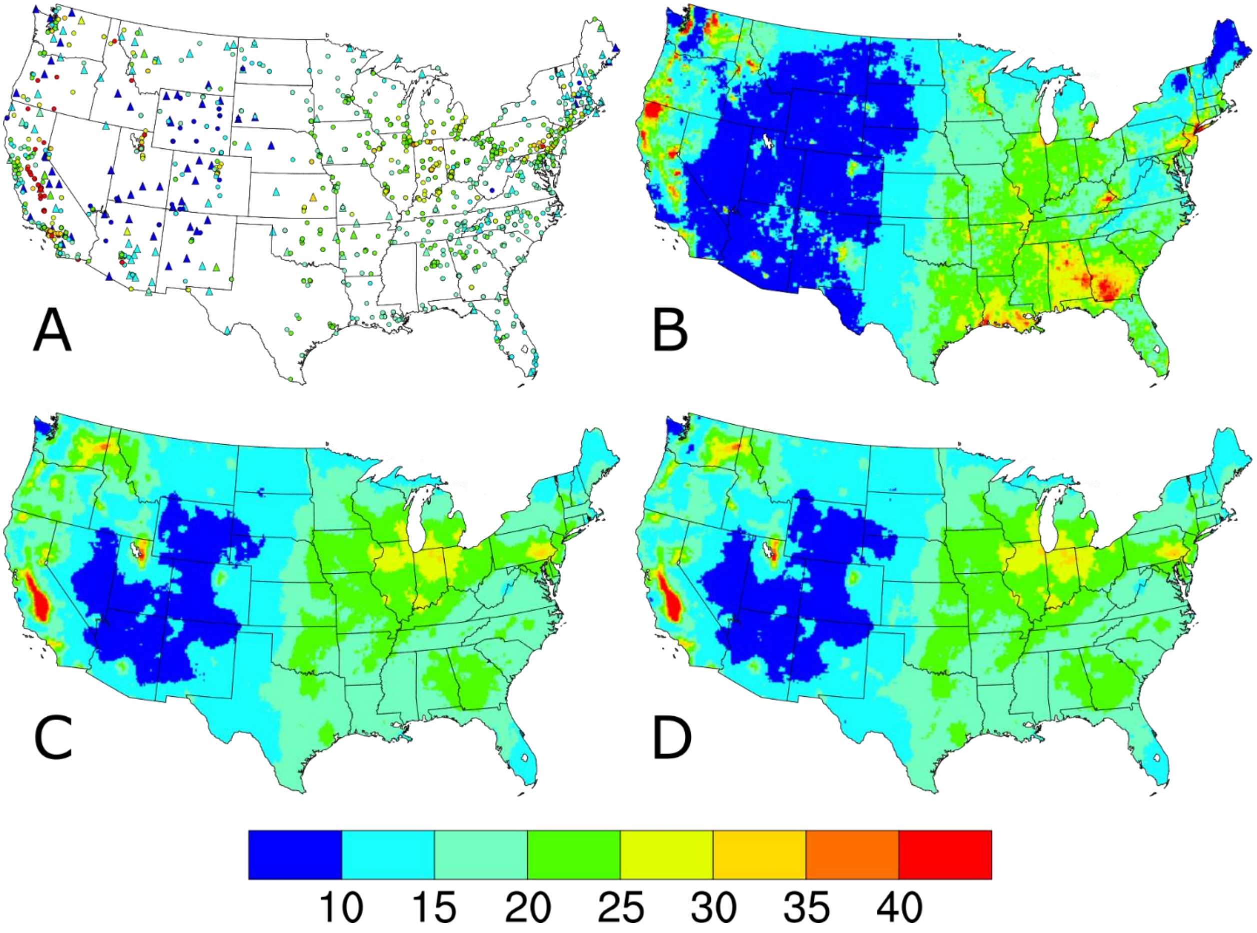


**Figure S2: $98^{th}$ percentile $PM_{2.5}$ concentrations (in units of $\mu g\ m^{-3}$) a) observed at the AQS (circles) and IMPROVE (triangles) sites and predicted for the entire period by the b) CMAQ model, c) Downscaler with the baseline configuration, and d) Downscaler with IMPROVE data. Note that the color scales differ across Figure S1 (annual averages) and Figure S2.**

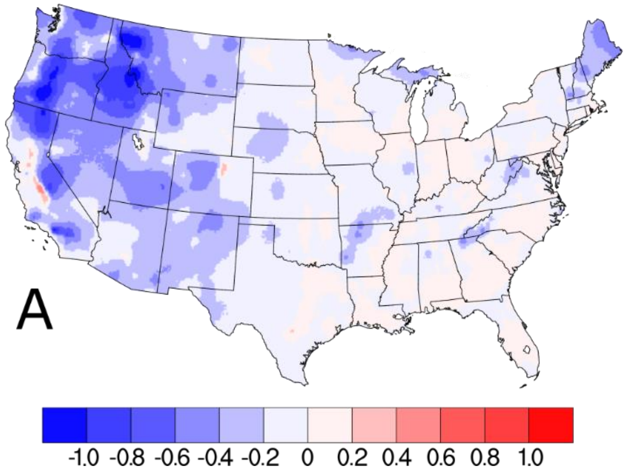


**Figure S3: Difference in Downscaler-predicted a) annual average and b) 98th percentile $PM_{2.5}$ concentrations (in units of μg $m^{-3}$) for the entire period between the w/IMPROVE and Baseline model runs. The values in these figures are calculated as w/IMPROVE – Baseline.**

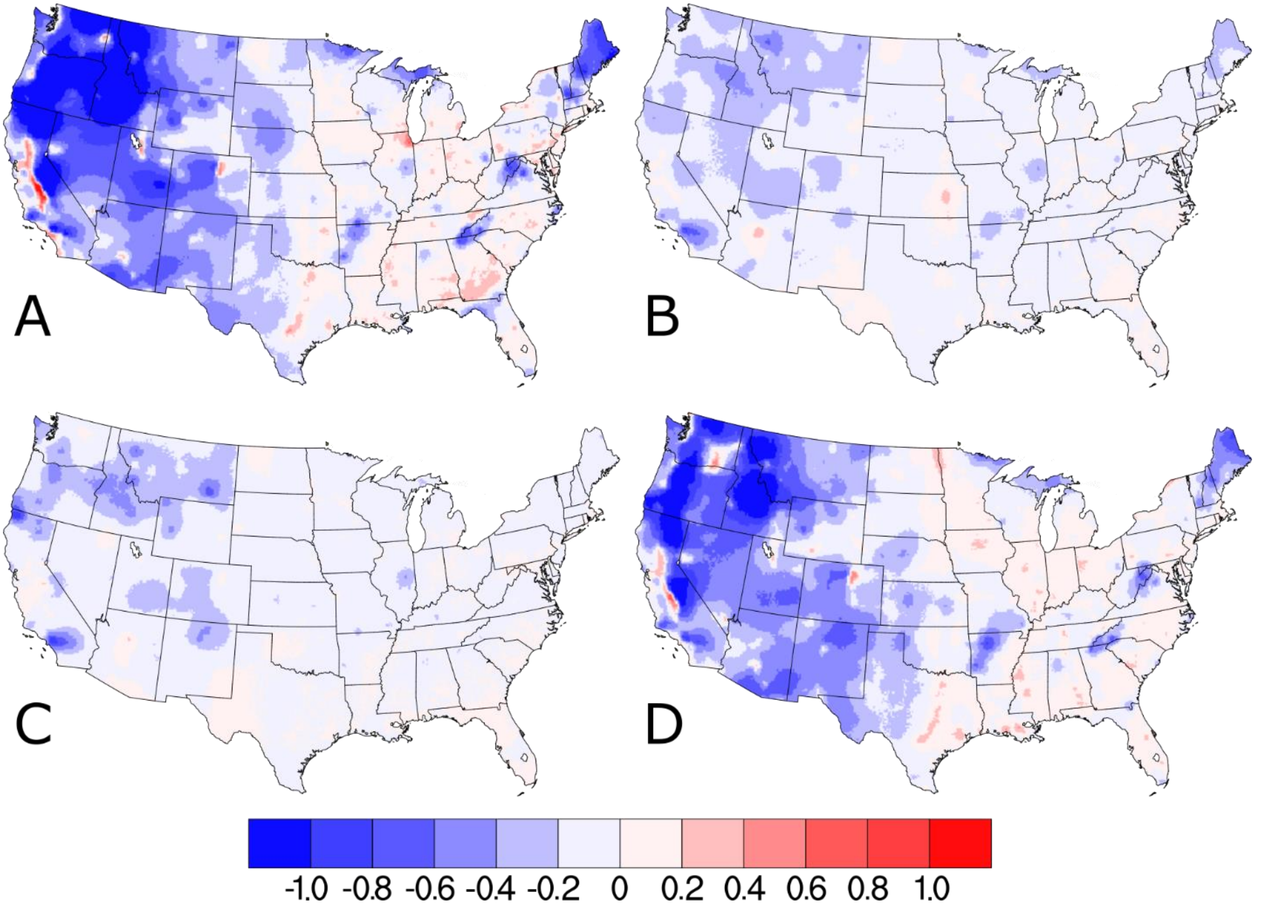


**Figure S4: Difference in Downscaler-predicted quarterly average $PM_{2.5}$ concentrations (in units of μg $m^{-3}$) for the entire period between the w/IMPROVE and Baseline model runs for a) Q1 (January, February, March), b) Q2 (April, May, June), c) Q3 (July. August, September), and d) Q4 (October, November, December). The values in these figures are calculated as w/IMPROVE – Baseline.**

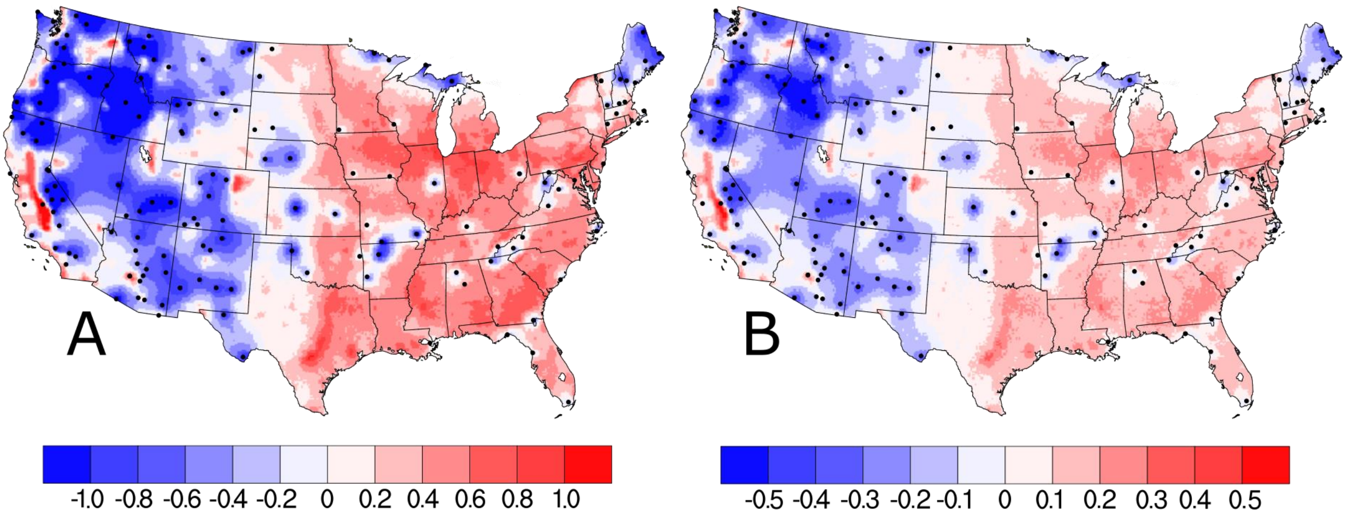


**Figure S5: Difference in Downscaler-predicted standard error in annual average $PM_{2.5}$ concentrations (in units of $\mu g\ m^{-3}$) for a) IMPROVE sample dates and b) the entire period between the w/IMPROVE and Baseline model runs. The values in these figures are calculated as w/IMPROVE – Baseline. The black dots indicate locations of the IMPROVE sites.**

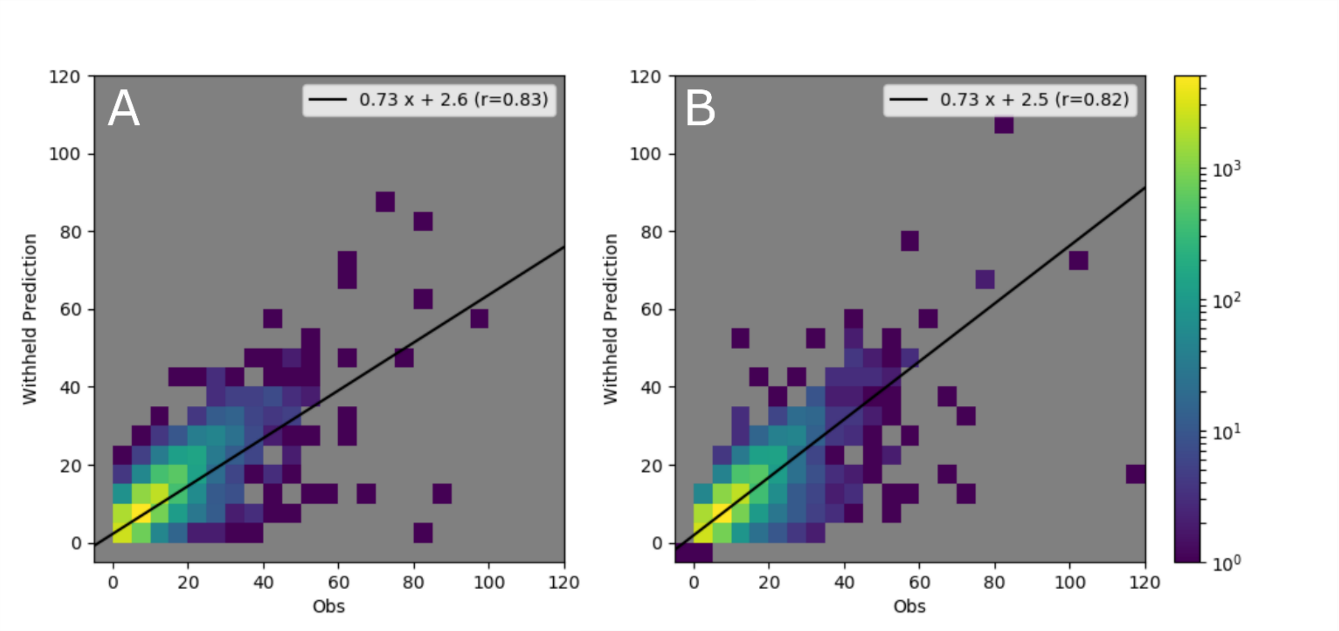


**Figure S6: Scatter density of the Downscaler validation (10% of data withheld) for AQS sites using a) the baseline configuration and b) including IMPROVE data. The x-axis values represent the average of the observed concentration for each AQS site-day withheld and the y-axis values represent the validation bias value (converted to concentration).**
